# Social anxiety intervention for college students: the effect of Dancesport exercise prescriptions combined with group psychological counseling

**DOI:** 10.3389/fpsyg.2026.1854500

**Published:** 2026-10-07

**Authors:** Mengmeng Yuan, Chen Jie, Dan Gong, Shunli Wu, Weikai Chen, Yuanning Li

**Affiliations:** 1 School of Marxism, Huainan Normal University, Huainan, Anhui, China; 2 School of Politics and Public Administration, Guangxi Normal University, Guilin, China; 3 Department of Student Affairs, Jiaxing Vocational Technical College, Jiaxing, China; 4 Chongqing College of International Business and Economics, College of Physical Education and Health, Chongqing, China; 5 College of Marine Life Sciences, Ocean University of China, Qingdao, China; 6 Department of Orthopedics, The Second Affiliated Hospital and Yuying Children’s Hospital of Wenzhou Medical University, Wenzhou, China; 7 School of Physical Education, Yanshan University, Qinhuangdao, Hebei, China

**Keywords:** dance therapy, exercise therapy, psychotherapy, randomized controlled trial, social anxiety disorder

## Abstract

**Objective:**

This randomized controlled trial (RCT) was designed to evaluate the relative efficacy of a structured dancesport exercise prescription, group psychological counseling, and their combined application in reducing social anxiety symptoms among Chinese university students.

**Methods:**

Forty-eight university students with elevated social anxiety were recruited from the Dancesport Club of Hefei Normal University and randomly allocated to four parallel groups (*n* = 12 per group): a Dancesport Exercise Prescription group (DP), a Group Psychological counseling group (GC), a Combined Intervention group (DP-GC), and a no-intervention control group. Social anxiety and related psychological symptoms were assessed before and after the 12-week intervention using the Symptom Checklist-90 (SCL-90), the Interaction Anxiousness Scale (IAS), the Social Avoidance and Distress Scale (SAD), and the Liebowitz Social Anxiety Scale (LSAS). Group differences over time were examined using a 4 × 2 Group × Time repeated-measures framework, supplemented by adjusted *post hoc* comparisons, effect-size estimates, and 95% confidence intervals.

**Results:**

The three active intervention conditions showed greater pre-to-post reductions in social anxiety-related outcomes than the control condition. The combined DP-GC group showed the largest improvement across the measured outcomes. However, because the combined condition integrated two intervention components within the same scheduled sessions and the sample size was small, the observed advantage is interpreted cautiously as a potential additive benefit rather than definitive evidence of a true synergistic interaction.

**Conclusion:**

Dancesport exercise prescription and group psychological counseling may each reduce social anxiety symptoms among college students, with complementary scale-specific effects. The combined intervention model appears promising, but its superiority should be interpreted cautiously in light of the integrated intervention design, small sample size, and reliance on self-report outcomes.

## 1 Introduction

Interpersonal communication constitutes a core developmental mechanism underpinning social adaptation and personality consolidation during late adolescence and emerging adulthood (Jimenez and Morreale, 2015). University students, situated at a critical juncture of self-concept formation, exhibit an intensified need for meaningful social connection and peer validation (Li et al., 2012). However, a converging body of epidemiological evidence indicates that social anxiety has surfaced as a predominant source of psychological morbidity within this demographic, positioning it as a central concern for contemporary developmental and clinical research (Tan et al., 2023; Al Hadid et al., 2023).

Social anxiety is a prevalent mental health condition defined by marked and persistent fear or apprehension regarding social or performance situations in which the individual is exposed to potential scrutiny by others (Morrison and Heimberg, 2013). Left unmitigated, it exerts a deleterious influence on interpersonal functioning, academic attainment, and overall quality of life (Mithawala and Thomas, 2021). Furthermore, subclinical manifestations frequently escalate to a formally diagnosable social anxiety disorder (SAD) during the college years (Purdon et al., 2001; Walder et al., 2023). Chronic social anxiety and SAD are robustly associated with enduring impairments across multiple life domains, including psychological well-being, somatic health, and long-term occupational trajectory (Luan et al., 2022). This burden underscores the imperative for developing accessible, efficacious early intervention strategies tailored to the collegiate population.

Cognitive-behavioral therapy (CBT) remains the first-line, empirically validated treatment for social anxiety, demonstrating well-established and stable therapeutic effects (O'Brien et al., 2007). However, the broader clinical implementation of CBT is frequently hampered by structural barriers, including prolonged treatment duration and substantial resource demands, which limit scalability within large university mental health systems (Miazgowski et al., 2021). In response to these constraints, group psychological counseling has emerged as a pragmatic and feasible alternative for addressing social anxiety among university students (Hu and Zhuang, 2007; Ueda and Tsuda, 2013; Yang et al., 2015). Extant research provides empirical support for its capacity to enhance interpersonal communication skills and attenuate social anxiety symptomatology. Nonetheless, the predominant focus of developmental group interventions in higher education has traditionally centered on career planning, with targeted protocols specifically designed to remediate social anxiety remaining comparatively underdeveloped. Moreover, while group counseling has demonstrated efficacy in improving interpersonal adaptability (Wang et al., 2020; Chakhssi et al., 2018), the durability of its therapeutic gains is contested; some investigations document persistent effects extending beyond two months, whereas others contend that benefits are immediate but transient (Stiles et al., 2015).

Concurrently, evidence indicates that physical exercise can reduce anxiety symptoms across different populations, including university students (Hou and Wang, 2021; Rebar et al., 2015; Stubbs et al., 2017; Lin and Gao, 2023; Singh et al., 2023). In the present study, dancesport is operationally defined as a structured, coach-led form of dance-based physical activity involving technical steps, rhythmic movement, and interpersonal coordination. The intervention program specifically incorporated Latin dance. Dancesport exercise refers to planned participation in these dance activities for the purposes of physical training and psychological intervention. Exercise prescription is an established concept in exercise science and is commonly structured around key components including frequency, intensity, time, type, volume, and progression (Li et al., 2024). Accordingly, the term “dancesport exercise prescription” in the present study refers to a structured Latin dance exercise program specifying exercise frequency, intensity, session duration, training content, and progression. As a multifaceted aerobic discipline integrating flexibility, balance, muscular endurance, and cardiovascular conditioning, dancesport has been associated with improvements in physical fitness and performance-related capacities (McCabe et al., 2013; Ren, 2023; Zhang et al., 2022; Liu et al., 2023). Beyond its physiological benefits, the structured musical accompaniment and rhythmic motor execution inherent in dancesport may alleviate somatic tension, facilitate affective regulation, and promote psychological well-being (McCabe et al., 2013; Liu et al., 2023; Yan et al., 2024). Specific dance modalities, particularly Latin dance, have also been associated with enhanced physical self-esteem and self-confidence and reduced social physique anxiety (Adilogullari, 2014; Liu et al., 2025).

Previous studies have reported benefits of dance-based interventions for anxiety-related outcomes (Adilogullari, 2014; Salihu et al., 2021; Liu et al., 2023; Yan et al., 2024; Liu et al., 2025; Leste and Rust, 1984), while group psychological counseling has been associated with improvements in interpersonal and social adaptation among students (Hu and Zhuang, 2007; Wang et al., 2020). However, limited research has integrated these two modalities within the same experimental paradigm to examine their combined and comparative effects among university students. A randomized controlled design is therefore useful for evaluating relative efficacy and exploring whether the combined approach produces an additional benefit beyond either intervention alone. Because the combined intervention integrates two intervention components within the same experimental condition, any apparent superiority should be interpreted with appropriate caution.

Social anxiety involves interconnected physiological, behavioral, cognitive, and affective processes, and interventions targeting only a single domain may not fully address its multidimensional symptom profile. This provides a theoretical basis for integrating intervention modalities that operate at different levels of the anxiety response. From a physiological and embodied-behavioral perspective, dancesport exercise may reduce somatic arousal and avoidance tendencies through moderate-intensity movement, rhythmic music, and repeated low-pressure interpersonal exposure (Lin and Gao, 2023; Salihu et al., 2021). In contrast, group psychological counseling may primarily support cognitive and affective adjustment through emotional expression, peer support, role-playing, and communication-skills practice (Bandura, 1977; Lattie et al., 2019). By addressing partially distinct components of social anxiety, the two interventions may complement one another, with dancesport providing embodied behavioral and interpersonal practice and group counseling offering structured psychological support. Their integration therefore provides a plausible theoretical and physiological basis for a broader, potentially additive intervention effect.

To address these gaps in empirical integration and theoretical grounding, the present study employed an RCT design to examine the effects of a dancesport exercise prescription, group psychological counseling, and their combined application on social anxiety in a cohort of Chinese university students. Forty-eight participants meeting the criteria for elevated social anxiety were recruited and allocated to four parallel arms to evaluate both standalone effects and the potential added benefit of a combined intervention. Validated psychometric instruments were administered at pre- and post-intervention to quantify changes in social anxiety severity.

The specific aims were: (1) to examine the standalone efficacy of dancesport exercise prescription and group psychological counseling; (2) to explore the potential added benefit of a combined intervention strategy; and (3) to provide a theoretically informed intervention framework for university-based mental health education.

## 2 Materials and methods

### 2.1 Participants

Initially, 150 students were recruited from the Dancesport Club of Hefei Normal University. Because the recruitment source was limited to a dancesport club, the sample should be understood as a convenience sample of students with interest in, or access to, dance-based physical activity rather than a representative sample of Chinese university students generally. All participants were undergraduate dancers recruited from the Dancesport Club. Given that they had been exposed to similar instructional curricula and training conditions, their dancesport proficiency was considered intermediate and generally comparable at baseline.

Prior to the experiment, all participants completed screening using the IAS, SAD, and LSAS. The inclusion criteria required that scores on all three social anxiety scales exceeded the respective normative subclinical thresholds (LSAS total score ≥ 31, IAS total score > 45, SAD total score > 14). Accordingly, 60 students who met the inclusion criteria were identified as potential intervention candidates. Ultimately, following the principle of voluntary participation, 48 students (24 males, 24 females; mean age = 21.13 ± 1.15 years) were formally enrolled and allocated to four parallel groups (*n* = 12 per group): the DP group, GC group, DP-GC group, and control group. *A priori* power analysis was conducted using G*Power 3.1 (Faul et al., 2007) for a repeated-measures ANOVA with a within-between interaction, assuming a medium effect size (*f* = 0.25), α = 0.05, and power = 0.80; the analysis estimated a required sample size of 48 participants (12 per group). All 48 allocated participants completed the 12-week intervention and were included in the final analysis. Group-level baseline demographic and clinical characteristics are summarized in Table 1.

**Table 1:** Baseline characteristics of participants by group.

Variable (M ± SD)	DP (*n* = 12)	GC (*n* = 12)	DP-GC	Control
Age (years)	21.15 ± 1.18	21.08 ± 1.12	21.17 ± 1.20	21.10 ± 1.14
Sex (male/female)	6/6	6/6	6/6	6/6
IAS pre-intervention	53.00 ± 3.51	52.88 ± 2.90	53.00 ± 3.74	52.62 ± 3.11
SAD pre-intervention	25.00 ± 1.31	24.88 ± 1.46	25.12 ± 1.13	25.12 ± 1.64
LSAS pre-intervention	89.38 ± 9.75	89.38 ± 12.45	88.88 ± 12.56	88.50 ± 13.87
SCL-90 anxiety pre-intervention	34.00 ± 6.21	33.75 ± 6.48	33.75 ± 4.46	34.25 ± 5.85

### 2.2 Measurement tools

Four validated psychometric scales were used to assess social anxiety and associated psychological symptoms at pre- and post-intervention. The scales were selected to capture affective, cognitive, behavioral, and broader psychological distress dimensions related to social anxiety. Because all primary outcomes were based on self-report instruments, potential response bias and social desirability effects should be considered when interpreting the findings.

#### 2.2.1 Symptom checklist 90 (SCL-90)

Developed by Derogatis (1975), the SCL-90 is a 90-item self-report scale designed to assess a broad range of psychological and somatic symptoms experienced over the preceding week (Gomez et al., 2021). It comprises nine primary dimensions: somatization, obsessive-compulsive symptoms, interpersonal sensitivity, depression, anxiety, hostility, phobic anxiety, paranoid ideation, and psychoticism, with additional items for general distress. Responses are rated on a 5-point Likert scale (1 = not at all, 5 = extremely severe). In the present study, the SCL-90 was used as a broad psychological distress measure rather than as a diagnosis-specific measure of social anxiety. Its hostility, paranoid ideation, psychoticism, anxiety, and interpersonal sensitivity dimensions were interpreted as auxiliary indicators of broader distress and social threat-related psychological experience in a subclinical college student sample (Xin et al., 2020) (Supplementary Appendix C).

#### 2.2.2 Interaction anxiousness scale (IAS)

The IAS, developed by Leary (1983), is a 15-item self-report scale that quantifies subjective feelings of unease, stress, and fear in social interaction scenarios, as well as negative cognitions about such interactions (Nichols and Webster, 2015). Distinct from behavioral measures of social anxiety, the IAS focuses on the affective and cognitive components of social anxiety tendencies and exhibits high internal consistency and construct validity. Responses are rated on a 5-point Likert scale, with total scores ranging from 15 (minimal social anxiety) to 75 (severe social anxiety). The scale is a gold-standard measure for assessing social interaction anxiety in non-clinical populations (Zhang et al., 2015) (Supplementary Appendix D).

#### 2.2.3 Social avoidance and distress scale (SAD)

Developed by Watson and Friend (1969), the SAD is a 28-item scale that assesses subjective experiences of social avoidance and distress across a variety of social contexts, including unfamiliar situations, being observed by others, public speaking, and casual conversation (Hofmann et al., 2004). The scale is divided into two subscales (14 items each) measuring social avoidance and social distress, with a binary scoring system (0 = no, 1 = yes). Total scores range from 0 (no social avoidance/distress) to 28 (severe social avoidance/distress), with higher scores indicating greater impairment. The SAD has been extensively validated for use in college student populations and is a key measure for evaluating behavioral and affective components of social anxiety (Turner et al., 1987) (Supplementary Appendix E).

#### 2.2.4 Liebowitz social anxiety scale (LSAS)

Introduced by Liebowitz (1987), the LSAS is a 24-item self-report scale that assesses the severity of anxiety and avoidance behaviors in social and performance situations (Mennin et al., 2002). It uses a 4-point Likert scale (0 = none/never to 3 = severe/always) to rate both anxiety severity and avoidance frequency for each item, with two subscales (Social Anxiety and Social Avoidance) and a total score ranging from 0 to 144 (0–72 for each subscale). The LSAS has well-established cut-off scores for social anxiety severity: 0–30 (normal), 31–60 (mild), 61–90 (moderate), and 91–144 (severe) (Baroni et al., 2023). As one of the most widely used measures of social anxiety, it demonstrates excellent reliability and validity for both clinical and non-clinical populations (Supplementary Appendix F).

### 2.3 Procedure

The study adopted a pre-test-post-test randomized controlled design, with all procedures conducted in accordance with the ethical guidelines of the Declaration of Helsinki and approved by the Institutional Review Board of Hefei Normal University. Participant recruitment, intervention implementation, and primary data collection were conducted at Hefei Normal University. Prior to study commencement, all participants received a detailed explanation of the study objectives, procedures, potential risks, and benefits, and provided written informed consent. Participants were informed of their right to withdraw from the study at any time without penalty, and all personal and assessment data were kept strictly confidential with de-identification for data analysis. Participants were allocated to the four groups in a 1:1:1:1 ratio using a randomization sequence generated by the RAND function in Microsoft Excel. The sequence was generated by a researcher who was not involved in participant recruitment or outcome assessment. No blocking or stratification was applied, and allocation was not concealed from the investigators. The lack of allocation concealment and stratification is acknowledged as a methodological limitation.

### 2.4 Intervention

The intervention lasted for 12 weeks, with the three intervention groups receiving structured interventions and the control group maintaining regular university activities (Supplementary Appendices A, B). The structured intervention schedule was organized as two sessions per week for each active intervention group. The DP group received two dancesport sessions per week, the GC group received two group psychological counseling sessions per week, and the DP-GC group received two integrated combined-intervention sessions per week. In the DP-GC group, components of the dancesport exercise prescription and group psychological counseling were delivered within the same scheduled sessions. The dancesport program comprised Latin dance. During the informed-consent process, participants in the DP and DP-GC groups were instructed to limit their dancesport participation to the sessions specified in the study protocol, whereas participants in the GC and control groups were instructed not to engage in dancesport during the 12-week intervention. Compliance with this requirement and habitual physical activity were not objectively monitored.

1. DP group: participants engaged in twice-weekly dancesport sessions under the supervision of a professional dancesport coach. The target exercise intensity was prescribed at 70–80% of age-predicted maximum heart rate (HRmax = 220 − age) (Martland et al., 2020). Because wearable heart-rate monitors were not used, adherence to this target range was not objectively verified. Each session was structured into three phases: preparatory warm-up (20 min), core dancesport training (30–50 min, progressively increased across the 12-week intervention), and post-exercise relaxation (15 min). The training progressed from basic posture, steps, and rhythmic movement to dance combinations, partner coordination, and the practice of complete routines in Latin dance.
2. GC group: participants attended twice-weekly group psychological counseling sessions led by nationally certified psychological counselors with extensive experience in group intervention for college students. The counseling curriculum was developed by the research team and focused on five core themes: Self-Discovery, Confidence Building, Self-Acceptance and Expressive Communication, Teamwork and Interpersonal Interaction, and Coping with Change and Social Adaptation. Sessions adopted a structured yet interactive format, including group discussion, role-playing, guided reflection, and peer support activities. The counseling progressed from self-awareness and confidence building to interpersonal interaction and social adaptation. Participants in this group did not receive the study-provided dancesport training.
3. DP-GC group: participants received an integrated combined-intervention program over 12 weeks. The program was delivered twice weekly, and each session incorporated components of dancesport exercise prescription and group psychological counseling. The dancesport component comprised Latin dance and followed the same training content, progression, target intensity, and exercise-session structure as the DP group.
4. Control group: participants received neither study-provided dancesport training nor group psychological counseling and maintained their regular academic and extracurricular activities throughout the 12-week period.

To maximize intervention adherence, the following strategies were implemented: regular check-ins and feedback collection to improve intervention delivery; proactive telephone follow-up for participants who missed four consecutive sessions; and peer support within the GC and DP-GC groups to promote participation and accountability. Attendance and protocol deviations were monitored throughout the 12-week intervention, and adherence information is summarized in Table 2. Pre-intervention assessments were conducted 1 week prior to the start of the intervention, and post-intervention assessments were completed within 3 days of the intervention conclusion. All scales were self-administered by participants in a standardized group setting under the supervision of trained personnel. The participant flow and overview of the four intervention conditions are presented in Figure 1.

**Table 2:** Participant completion and intervention adherence by group.

Group	Allocated	Completed	Mean sessions attended	≥80% attendance, *n* (%)	Protocol deviations
DP	12	12	24	100%	None
GC	12	12	24	100%	None
DP-GC	12	12	24	100%	None
Control	12	12	N/A	N/A	None

**Figure 1:**
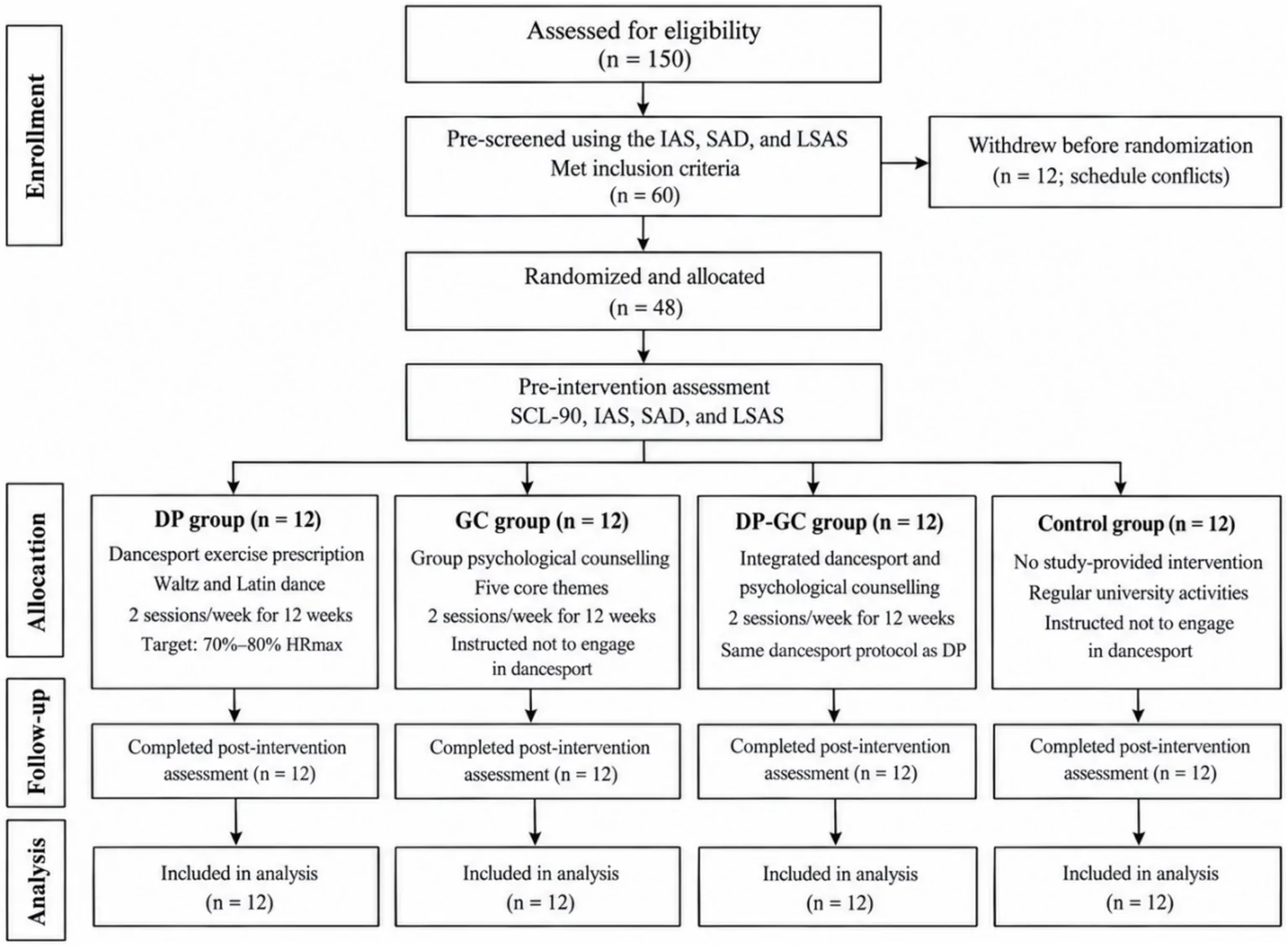
Participant flow and overview of the four intervention conditions.

### 2.5 Statistical analysis

All statistical analyses were performed using IBM SPSS Statistics version 26.0. Continuous variables are presented as mean ± standard deviation (M ± SD). The completeness and distributional characteristics of the data were examined before inferential analysis. Normality of model residuals was assessed using the Shapiro–Wilk test together with visual inspection of Q–Q plots. Homogeneity of variance across groups was evaluated using Levene’s test. Standardized residuals and boxplots were inspected to identify potential outliers and marked departures from model assumptions. Intervention effects were analyzed using a 4 × 2 mixed-design repeated-measures analysis of variance (ANOVA), with Group (DP, GC, DP-GC, and Control) specified as the between-subjects factor and Time (pre-intervention and post-intervention) as the within-subjects factor. The model evaluated the main effects of Group and Time and the Group × Time interaction. The Group × Time interaction was considered the principal effect of interest because it tested whether the magnitude of pre-to-post change differed among the four study groups. As Time contained only two measurement occasions, the sphericity assumption was not applicable. All randomized participants had complete pre- and post-intervention outcome data; therefore, no missing-data imputation was required.

Following identification of a significant Group × Time interaction, pairwise comparisons of pre-to-post changes were performed with Tukey adjustment to determine specific between-group differences. Partial eta squared (partial η^2^) was used to quantify ANOVA effect sizes. Mean differences (MDs), 95% confidence intervals (95% CIs), Cohen’s d, and adjusted *p* values (padj) were reported for pairwise comparisons. All statistical tests were two-sided, and statistical significance was defined as *p* < 0.05.

## 3 Results

### 3.1 Participant completion and adherence

Eligibility was assessed in 150 students, of whom 60 were found to meet the inclusion criteria. Prior to randomization, 12 eligible students withdrew because of conflicts with their academic schedules, resulting in 48 participants being allocated to the four study groups. The processes of screening, eligibility assessment, group allocation, follow-up, and final analysis are presented in the CONSORT-style flow diagram (Figure 1). The 12-week intervention protocol was completed by all 48 allocated participants, and all participants were subsequently included in the final analysis. Group-level attendance and protocol deviations are summarized in Table 2.

### 3.2 Symptom checklist 90

Beyond the primary measures of social anxiety, the effects of the four intervention conditions were further examined across a broader range of psychological symptoms using the nine SCL-90 subscales. Repeated-measures ANOVA revealed significant Group × Time interactions for all nine symptom dimensions (all *p* < 0.001), indicating that the magnitude of pre-to-post symptom change differed systematically across the four groups (Figure 2). The interaction effects were consistently substantial, with partial η^2^ values ranging from 0.43 to 0.58. Significant interactions were observed for Somatization, *F*(3, 44) = 18.4, partial η^2^ = 0.56; Obsessive-Compulsive symptoms, *F*(3, 44) = 12.9, partial η^2^ = 0.47; Interpersonal Sensitivity, *F*(3, 44) = 14.6, partial η^2^ = 0.50; Depression, *F*(3, 44) = 18.1, partial η^2^ = 0.55; Anxiety, *F*(3, 44) = 10.8, partial η^2^ = 0.43; Hostility, *F*(3, 44) = 13.7, partial η^2^ = 0.49; Phobic Anxiety, *F*(3, 44) = 15.2, partial η^2^ = 0.52; Paranoid Ideation, *F*(3, 44) = 17.3, partial η^2^ = 0.54; and Psychoticism, *F*(3, 44) = 19.0, partial η^2^ = 0.58.

**Figure 2:**
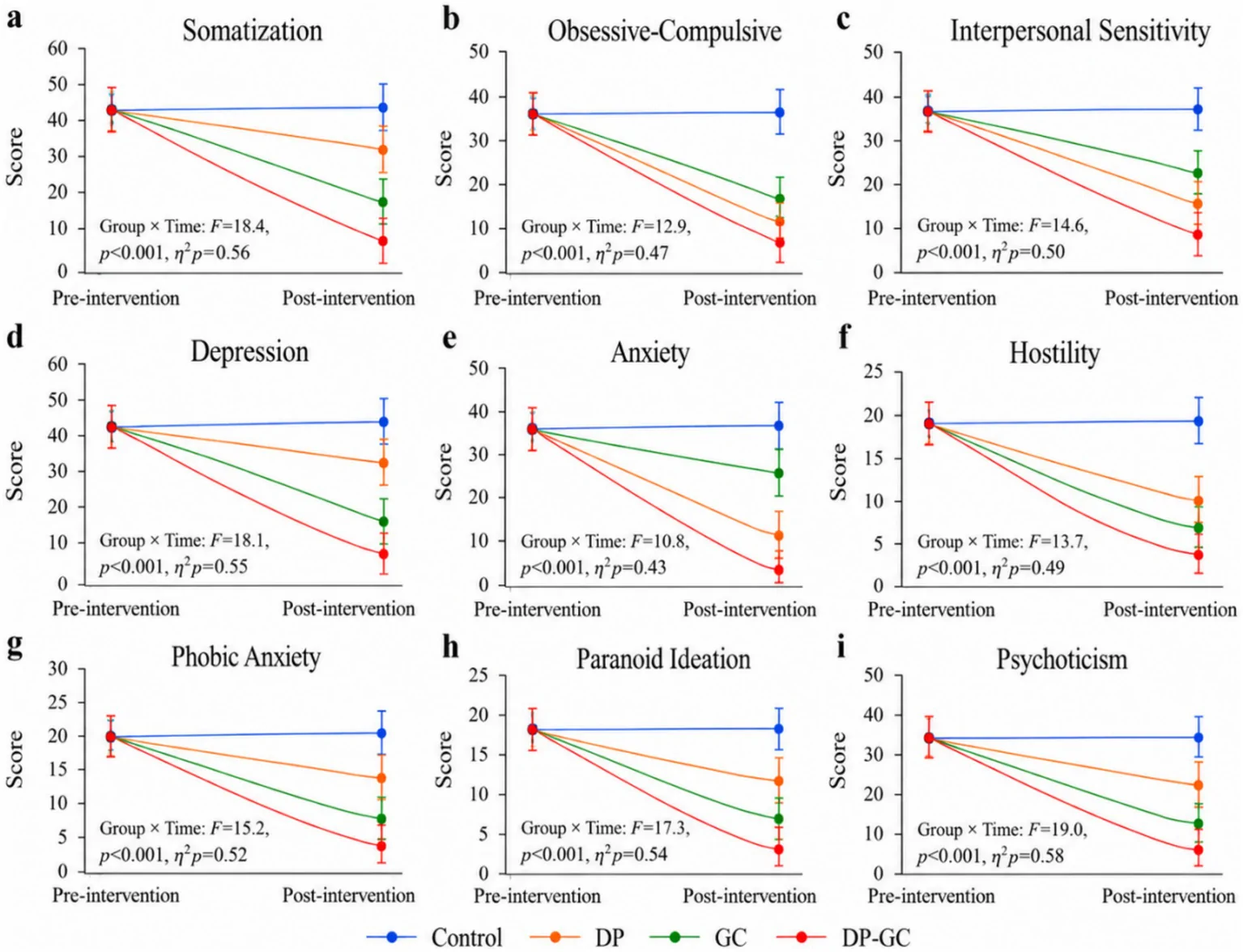
Changes in psychological symptom scores across different intervention groups before and after the intervention. **(a)** Somatization; **(b)** Obsessive-compulsive; **(c)** Interpersonal sensitivity; **(d)** Depression; **(e)** Anxiety; **(f)** Hostility; **(g)** Phobic anxiety; **(h)** Paranoid ideation; and **(i)** Psychoticism. Data are presented as mean ± SD.

Adjusted *post hoc* comparisons further demonstrated marked between-group differences in symptom reduction. For Somatization (Figure 2a), the DP-GC group showed the largest improvement (*M*change = 25.17), with significantly greater reductions than both the DP group (MD = 17.34, 95% CI [12.87, 21.81], Cohen’s *d* = 3.62, *p*adj < 0.001) and the GC group (MD = 7.59, 95% CI [3.42, 11.76], Cohen’s *d* = 1.72, *p*adj = 0.004). A similar pattern was observed for Obsessive-Compulsive symptoms (Figure 2b), with the DP-GC group showing greater improvement than the DP group (MD = 7.25, 95% CI [3.08, 11.42], Cohen’s *d* = 1.58, *p*adj = 0.008) and the GC group (MD = 6.42, 95% CI [2.25, 10.59], Cohen’s *d* = 1.49, *p*adj = 0.014). For Interpersonal Sensitivity (Figure 2c), the DP-GC condition likewise produced a larger reduction than the DP group (MD = 8.33, 95% CI [4.16, 12.50], Cohen’s *d* = 1.92, *p*adj < 0.001) and the GC group (MD = 6.17, 95% CI [1.99, 10.34], Cohen’s *d* = 1.38, *p*adj = 0.022). The same overall response pattern was evident for Depression (Figure 2d). The DP-GC group demonstrated the greatest reduction (*M*change = 23.33), significantly exceeding both the DP group (MD = 16.00, 95% CI [11.80, 20.20], Cohen’s *d* = 3.45, *p*adj < 0.001) and the GC group (MD = 7.16, 95% CI [2.96, 11.36], Cohen’s *d* = 1.58, *p*adj = 0.003). The GC group also showed greater improvement than the DP group (MD = 8.84, 95% CI [4.64, 13.04], Cohen’s *d* = 1.89, *p*adj < 0.001). In contrast, Anxiety exhibited a distinct response profile (Figure 2e). Although the DP-GC group again showed the largest reduction (*M*change = 24.83), its improvement exceeded that of the DP group by MD = 8.00 (95% CI [4.09, 11.91], Cohen’s *d* = 1.98, *p*adj < 0.001) and that of the GC group by MD = 19.00 (95% CI [15.09, 22.91], Cohen’s *d* = 4.70, *p*adj < 0.001). The DP group also demonstrated substantially greater improvement than the GC group (MD = 11.00, 95% CI [7.09, 14.91], Cohen’s *d* = 2.72, *p*adj < 0.001), suggesting a symptom-specific advantage of dancesport exercise for anxiety-related manifestations. For Hostility (Figure 2f), the DP-GC group showed the largest reduction (*M*change = 14.75), with greater improvement than the DP group (MD = 7.50, 95% CI [4.10, 10.90], Cohen’s *d* = 2.12, *p*adj < 0.001) and the GC group (MD = 5.50, 95% CI [2.10, 8.90], Cohen’s *d* = 1.56, *p*adj = 0.003). The difference between the GC and DP groups was comparatively modest (MD = 2.00, 95% CI [−1.40, 5.40], Cohen’s *d* = 0.57, *p*adj = 0.36). A comparable pattern was observed for Phobic Anxiety (Figure 2g), for which the DP-GC group achieved greater reductions than the DP group (MD = 8.66, 95% CI [5.10, 12.22], Cohen’s *d* = 2.32, *p*adj < 0.001) and the GC group (MD = 5.75, 95% CI [2.19, 9.31], Cohen’s *d* = 1.54, *p*adj = 0.004). The difference between GC and DP was smaller and did not remain statistically significant after adjustment (MD = 2.91, 95% CI [−0.65, 6.47], Cohen’s *d* = 0.78, *p*adj = 0.14). For Paranoid Ideation (Figure 2h), the DP-GC group again exhibited the greatest reduction (*M*change = 14.67), significantly exceeding the DP group (MD = 8.17, 95% CI [4.73, 11.61], Cohen’s *d* = 2.25, *p*adj < 0.001) and the GC group (MD = 4.34, 95% CI [0.90, 7.78], Cohen’s *d* = 1.20, *p*adj = 0.018). The GC group also showed a greater reduction than the DP group (MD = 3.83, 95% CI [0.39, 7.27], Cohen’s *d* = 1.06, *p*adj = 0.028). Finally, for Psychoticism (Figure 2i), the DP-GC group demonstrated the largest improvement (*M*change = 22.75), with significantly greater reductions than both the DP group (MD = 13.50, 95% CI [9.36, 17.64], Cohen’s *d* = 3.07, *p*adj < 0.001) and the GC group (MD = 5.83, 95% CI [1.69, 9.97], Cohen’s *d* = 1.32, *p*adj = 0.009). The GC group also outperformed the DP group (MD = 7.67, 95% CI [3.53, 11.81], Cohen’s *d* = 1.74, *p*adj < 0.001).

Across all nine SCL-90 dimensions, symptom scores remained largely stable in the control group, whereas the active intervention groups exhibited clear post-intervention reductions. Collectively, the consistently large interaction effects and moderate-to-very-large pairwise effect sizes indicate that the intervention-related changes extended beyond the core features of social anxiety to encompass a broad spectrum of psychological symptoms. Both dancesport exercise prescription and group psychological counseling were associated with multidimensional psychological improvement, although their relative effects varied across specific symptom domains. The DP-GC condition produced the most consistently pronounced reductions across the SCL-90 subscales. Nevertheless, because the present design did not isolate the independent and interactive effects of the two intervention components, the superior outcomes of the DP-GC condition should be interpreted as evidence of a potentially additive benefit rather than definitive proof of a synergistic interaction between dancesport exercise and group psychological counseling.

### 3.3 Interaction anxiousness scale

To further evaluate the effects of the different intervention conditions on interaction anxiety, pre-to-post changes in Interaction Anxiousness Scale (IAS) scores were compared across the four groups. Repeated-measures ANOVA revealed a significant Group × Time interaction, *F*(3, 44) = 25.3, *p* < 0.001, partial η^2^ = 0.63, indicating that the magnitude of change in interaction anxiety differed substantially among the intervention conditions (Figure 3). Adjusted *post hoc* comparisons indicated that all three active intervention groups achieved significantly greater reductions in IAS scores than the control group (all adjusted ps ≤ 0.007), whereas the control group showed only minimal change (Mchange = 1.00). The DP-GC group exhibited the largest reduction (Mchange = 30.50), significantly exceeding the improvement observed in the DP group (Mchange = 13.00; MD = 17.50, 95% CI [8.16, 26.84], Cohen’s d = 2.04, padj < 0.001) and the GC group (Mchange = 20.50; MD = 10.00, 95% CI [0.66, 19.34], Cohen’s d = 1.17, padj = 0.032). Although the GC group showed a numerically greater reduction than the DP group, the between-group difference did not reach statistical significance after adjustment (MD = 7.50, 95% CI [−1.84, 16.84], Cohen’s d = 0.88, padj = 0.155). The essentially stable IAS trajectory observed in the control group further suggests that the reductions evident in the active intervention conditions were unlikely to reflect spontaneous improvement over time. Collectively, these findings indicate that both dancesport exercise prescription and group psychological counseling were associated with meaningful reductions in interaction anxiety, while the combined DP-GC condition produced the greatest overall improvement. The large Group × Time interaction effect and the substantial pairwise effect sizes further suggest a potentially additive benefit of integrating the two intervention approaches.

**Figure 3:**
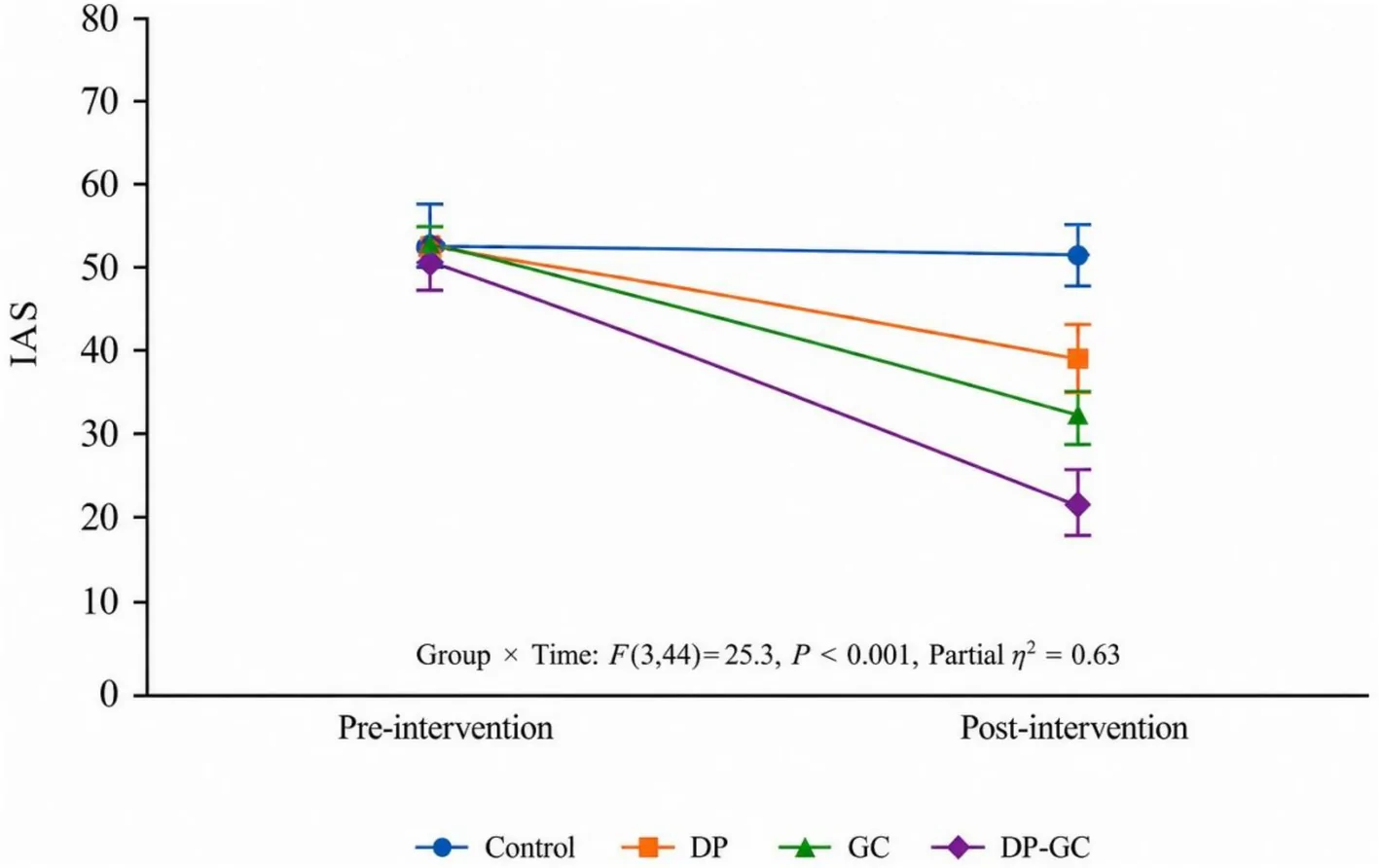
Changes in IAS scores across intervention groups before and after the intervention. The blue, orange, green, and purple lines represent the Control, DP, GC, and DP-GC groups, respectively. Data are presented as mean ± error bars.

### 3.4 Social avoidance and distress scale

The differential effects of the four intervention conditions were also evident in Social Avoidance and Distress (SAD) scores. Repeated-measures ANOVA revealed a significant Group × Time interaction, *F*(3, 44) = 25.3, *p* < 0.001, partial η^2^ = 0.63, indicating that the magnitude of pre-to-post change in social avoidance and distress differed substantially across the four groups (Figure 4). Tukey-adjusted *post hoc* comparisons showed that all three active intervention groups achieved significantly greater reductions than the control group: DP versus Control (MD = 13.39, 95% CI [7.70, 19.08], Cohen’s d = 2.57, padj < 0.001), GC versus Control (MD = 8.89, 95% CI [3.20, 14.58], Cohen’s d = 1.70, padj < 0.001), and DP-GC versus Control (MD = 17.72, 95% CI [12.03, 23.41], Cohen’s d = 3.39, padj < 0.001). The DP-GC group exhibited the largest reduction in SAD scores (Mchange = 17.50) and showed significantly greater improvement than the GC group (MD = 8.83, 95% CI [3.14, 14.52], Cohen’s d = 1.69, padj < 0.001). Although the reduction in the DP-GC group was numerically greater than that in the DP group (MD = 4.33, 95% CI [−1.36, 10.02], Cohen’s d = 0.83), this difference did not remain statistically significant after adjustment (padj = 0.192). Similarly, the DP group showed a numerically greater reduction than the GC group (MD = 4.50, 95% CI [−1.19, 10.19], Cohen’s d = 0.86), but the adjusted comparison was not statistically significant (padj = 0.165). In contrast, the control group showed minimal pre-to-post change, consistent with the essentially stable trajectory illustrated in Figure 4. Taken together, these findings demonstrate substantial intervention-related improvements in social avoidance and distress, with the combined DP-GC condition producing the largest overall reduction. However, the nonsignificant adjusted difference between the two standalone interventions indicates that their relative effects on SAD should be interpreted cautiously and should not be taken as evidence of the general superiority of DP over GC across psychological outcomes.

**Figure 4:**
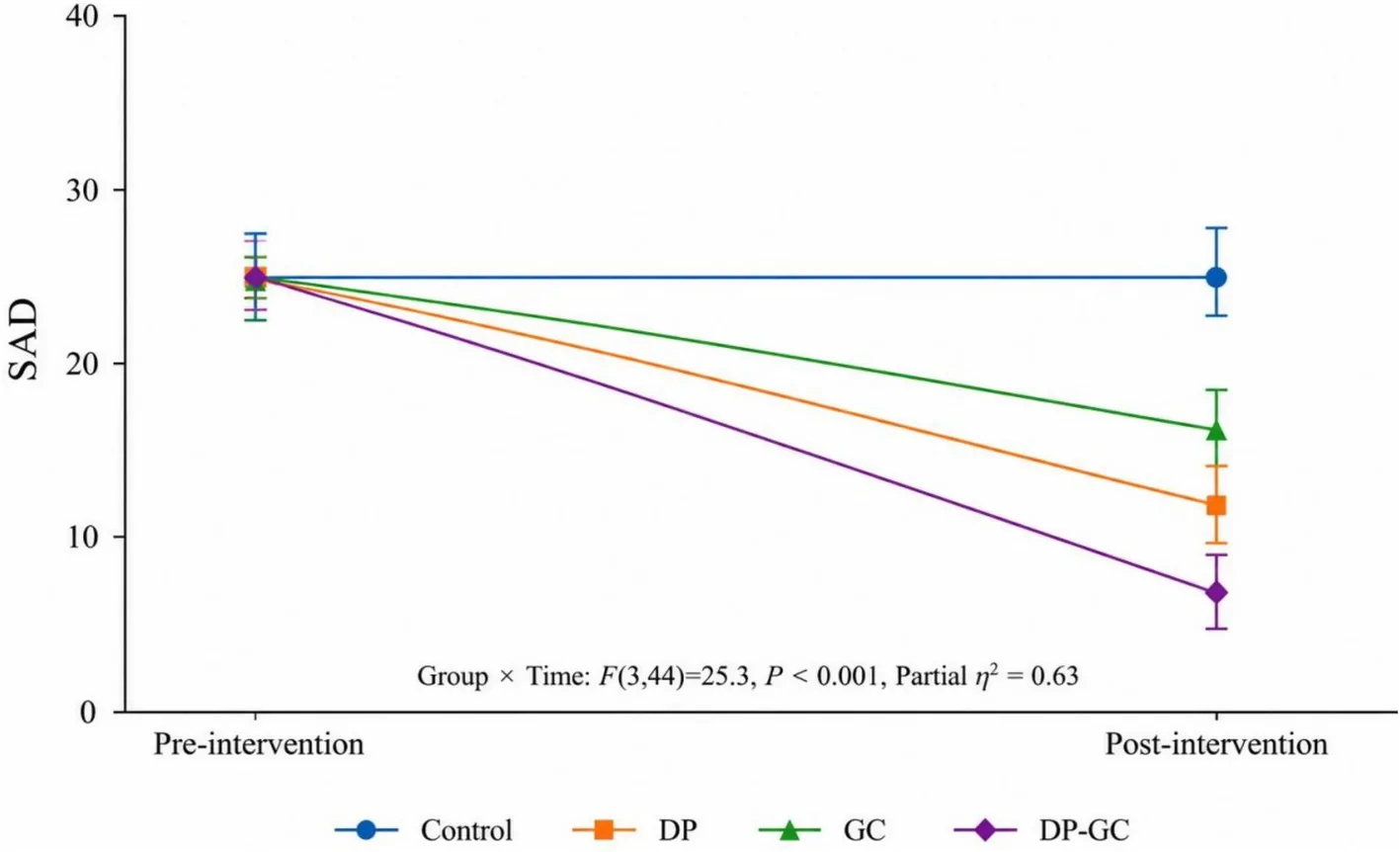
Changes in Social Avoidance and Distress (SAD) scores across the four groups from pre-intervention to post-intervention. The DP-GC group exhibited the greatest reduction in SAD scores, followed by the DP and GC groups, whereas the Control group remained largely unchanged.

### 3.5 Liebowitz social anxiety scale

To characterize changes in the overall severity of social anxiety, pre-to-post differences in Liebowitz Social Anxiety Scale (LSAS) scores were examined across the four study groups. Repeated-measures ANOVA revealed a significant Group × Time interaction, *F*(3, 44) = 58.71, *p* < 0.001, partial η^2^ = 0.800, indicating a substantial differential change in LSAS scores across the intervention period (Figure 5). Tukey-adjusted *post hoc* comparisons showed that all three active intervention groups achieved significantly greater reductions than the control group (all adjusted *ps* < 0.001). The DP-GC group exhibited the largest reduction in LSAS scores (*M*change = 39.70), significantly exceeding the improvement observed in the DP group (*M*change = 16.01; MD = 23.69, 95% CI [15.47, 31.91], Cohen’s *d* = 3.14, *p*adj < 0.001) and the GC group (*M*change = 27.61; MD = 12.08, 95% CI [3.87, 20.30], Cohen’s *d* = 1.60, *p*adj = 0.002). The GC group also demonstrated a significantly greater reduction than the DP group (MD = 11.61, 95% CI [3.39, 19.82], Cohen’s *d* = 1.54, *p*adj = 0.003). In contrast, the control group showed minimal change (*M*change = 0.61), consistent with the largely stable trajectory evident in Figure 5. Notably, the greater improvement observed in the GC group relative to the DP group on the LSAS contrasted with the response pattern identified for SAD scores. This divergence suggests that dancesport exercise prescription and group psychological counseling may exert differential, outcome-specific effects on distinct components of social anxiety, rather than one standalone intervention being uniformly superior across all measures. The consistently greater reduction observed in the DP-GC group further supports a potentially additive benefit of combining the two intervention approaches.

**Figure 5:**
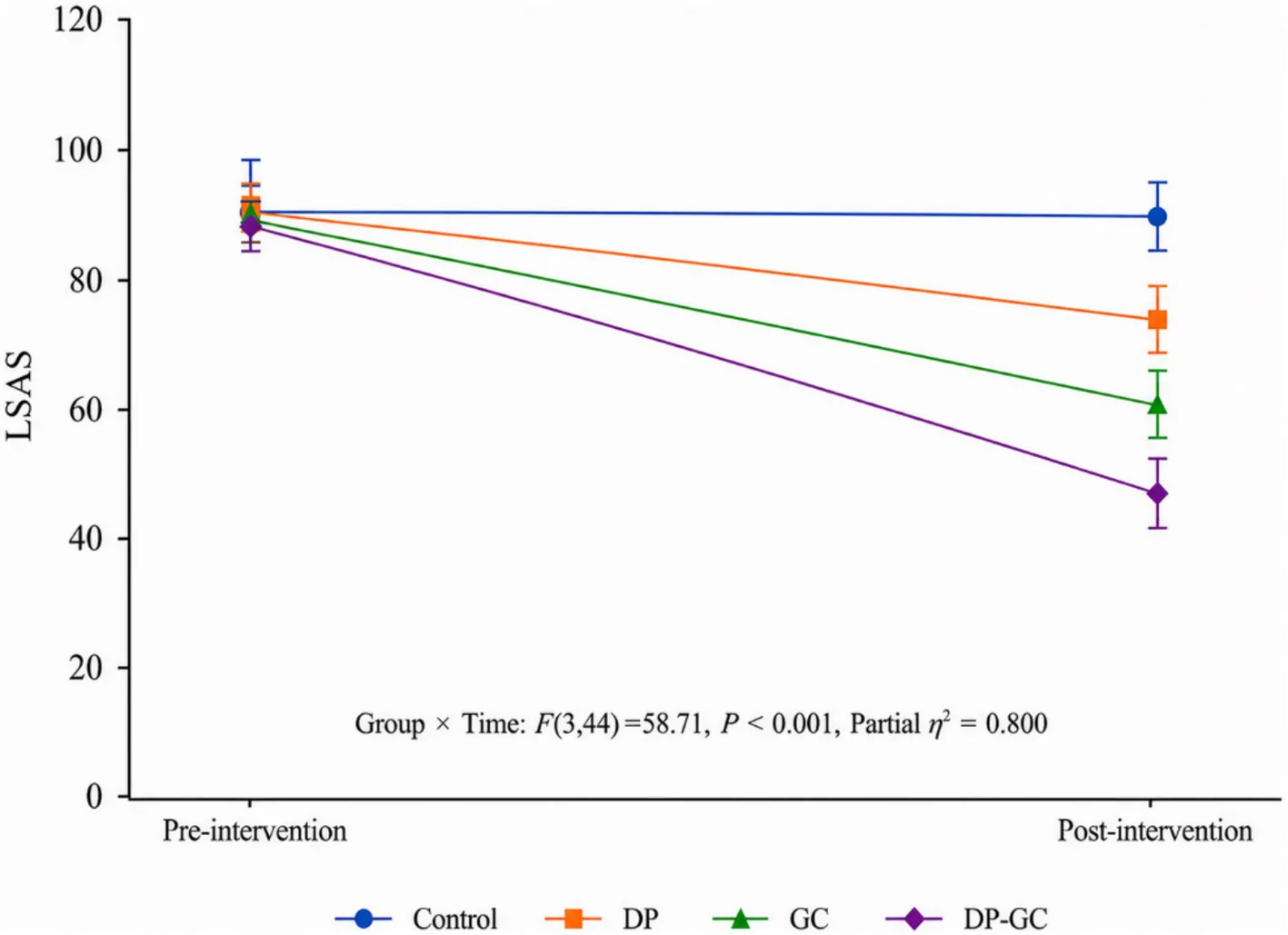
Changes in Liebowitz Social Anxiety Scale (LSAS) scores across the four groups from pre-intervention to post-intervention.

As summarized in Table 3, significant Group × Time interactions were observed across all outcome measures, including the nine SCL-90 dimensions, IAS, SAD, and LSAS, with all interaction effects reaching *p* < 0.001. Across the SCL-90 dimensions, the DP-GC group showed the largest reductions and demonstrated significantly greater improvement than both the DP and GC groups in all nine subscales, with adjusted comparisons ranging from *p* < 0.001 to *p* = 0.022. Comparisons between the two standalone interventions varied by symptom domain: GC showed significantly greater improvement than DP for Depression (*p* < 0.001), Paranoid Ideation (*p* = 0.028), and Psychoticism (*p* < 0.001), whereas DP showed significantly greater improvement than GC for Anxiety (*p* < 0.001). No significant GC–DP differences were observed for Hostility (*p* = 0.360) or Phobic Anxiety (*p* = 0.140).

**Table 3:** Overview of pre-to-post changes and Tukey-adjusted *post hoc* comparisons across outcome measures.

Outcome	Pre-to-post pattern	Group × Time interaction	Tukey-adjusted *post hoc* comparisons of pre-to-post changes
SCL-90 Somatization	DP-GC showed the largest reduction	***F*(3,44) = 18.4; *p* < 0.001; partial η**^**2**^ **= 0.56**	**DP-GC > DP, *p* < 0.001; DP-GC > GC, *p* = 0.004**
SCL-90 Obsessive-Compulsive	DP-GC showed the largest reduction	***F*(3,44) = 12.9; *p* < 0.001; partial η**^**2**^ **= 0.47**	**DP-GC > DP, *p* = 0.008; DP-GC > GC, *p* = 0.014**
SCL-90 Interpersonal Sensitivity	DP-GC showed the largest reduction	***F*(3,44) = 14.6; *p* < 0.001; partial η**^**2**^ **= 0.50**	**DP-GC > DP, *p* < 0.001; DP-GC > GC, *p* = 0.022**
SCL-90 Depression	DP-GC showed the largest reduction	***F*(3,44) = 18.1; *p* < 0.001; partial η**^**2**^ **= 0.55**	**DP-GC > DP, *p* < 0.001; DP-GC > GC, *p* = 0.003; GC > DP, *p* < 0.001**
SCL-90 Anxiety	DP-GC showed the largest reduction; DP > GC	***F*(3,44) = 10.8; *p* < 0.001; partial η**^**2**^ **= 0.43**	**DP-GC > DP, *p* < 0.001; DP-GC > GC, *p* < 0.001; DP > GC, *p* < 0.001**
SCL-90 Hostility	DP-GC showed the largest reduction	***F*(3,44) = 13.7; *p* < 0.001; partial η**^**2**^ **= 0.49**	**DP-GC > DP, *p* < 0.001; DP-GC > GC, *p* = 0.003;** GC vs. DP, *p* = 0.360
SCL-90 Phobic Anxiety	DP-GC showed the largest reduction	***F*(3,44) = 15.2; *p* < 0.001; partial η**^**2**^ **= 0.52**	**DP-GC > DP, *p* < 0.001; DP-GC > GC, *p* = 0.004;** GC vs. DP, *p* = 0.140
SCL-90 Paranoid Ideation	DP-GC showed the largest reduction	***F*(3,44) = 17.3; *p* < 0.001; partial η**^**2**^ **= 0.54**	**DP-GC > DP, *p* < 0.001; DP-GC > GC, *p* = 0.018; GC > DP, *p* = 0.028**
SCL-90 Psychoticism	DP-GC showed the largest reduction	***F*(3,44) = 19.0; *p* < 0.001; partial η**^**2**^ **= 0.58**	**DP-GC > DP, *p* < 0.001; DP-GC > GC, *p* = 0.009; GC > DP, *p* < 0.001**
IAS	DP-GC > GC; DP-GC > DP; Control minimal change	***F*(3,44) = 25.3; *p* < 0.001; partial η**^**2**^ **= 0.63**	**DP, GC, and DP-GC > Control, all *p* ≤ 0.007; DP-GC > DP, *p* < 0.001; DP-GC > GC, *p* = 0.032;** GC vs. DP, *p* = 0.155
SAD	DP-GC > GC; Control minimal change	***F*(3,44) = 25.3; *p* < 0.001; partial η**^**2**^ **= 0.63**	**DP > Control, *p* < 0.001; GC > Control, *p* < 0.001; DP-GC > Control, *p* < 0.001; DP-GC > GC, *p* < 0.001;** DP-GC vs. DP, *p* = 0.192; DP vs. GC, *p* = 0.165
LSAS	DP-GC > GC > DP; Control minimal change	***F*(3,44) = 58.71; *p* < 0.001; partial η**^**2**^ **= 0.800**	**DP, GC, and DP-GC > Control, all *p* < 0.001; DP-GC > DP, *p* < 0.001; DP-GC > GC, *p* = 0.002; GC > DP, *p* = 0.003**

Bold values indicate statistically significant results (*p* < 0.05).

For the social anxiety-specific outcomes, all three active intervention groups showed significantly greater reductions than the control group on IAS, SAD, and LSAS. The DP-GC group showed significantly greater improvement than both standalone groups on IAS, exceeding DP (*p* < 0.001) and GC (*p* = 0.032). For SAD, DP-GC showed significantly greater improvement than GC (*p* < 0.001), but its difference from DP was not statistically significant after Tukey adjustment (*p* = 0.192). For LSAS, the DP-GC group showed significantly greater improvement than both DP (*p* < 0.001) and GC (*p* = 0.002), and GC also showed a significantly greater reduction than DP (*p* = 0.003). Overall, Table 3 indicates that the combined DP-GC intervention produced the most consistent pattern of improvement, although the relative effects of the two standalone interventions differed across outcome measures.

## 4 Discussion

The present study examined the standalone and combined effects of dancesport exercise prescription and group psychological counseling on social anxiety-related outcomes among Chinese college students. Significant Group × Time interactions were observed for the IAS, SAD, and LSAS outcomes, as well as across the nine SCL-90 dimensions. The three active intervention conditions generally showed greater pre-to-post reductions than the control condition, whereas the DP-GC group demonstrated the largest improvement across most measured outcomes. These findings indicate that both intervention approaches were associated with short-term reductions in social anxiety-related symptoms and that the combined intervention produced the broadest pattern of improvement in the present sample.

The comparative findings for the two standalone interventions were not uniform across outcome measures. For IAS, the reduction observed in the GC group was numerically greater than that in the DP group, but the adjusted between-group difference was not statistically significant. A different numerical pattern was observed for SAD, for which the DP group showed a greater reduction than the GC group; however, this difference also did not remain statistically significant after adjustment. In contrast, the GC group demonstrated a significantly greater reduction in LSAS scores than the DP group. Taken together, these findings do not support the general superiority of either standalone intervention. Rather, the variation across outcome measures may indicate differential response patterns across dimensions of social anxiety. Because the psychological processes underlying these scale-specific differences were not directly measured, the findings should not be interpreted as evidence that either intervention selectively altered a specific cognitive, emotional, or behavioral mechanism.

One possible explanation for the observed intervention effects is that dancesport exercise and group psychological counseling may involve distinct but potentially complementary therapeutic processes. Moderate-intensity rhythmic movement and repeated interpersonal interaction during dancesport may provide behavioral engagement and social exposure in a relatively structured context, consistent with previous evidence concerning the anxiolytic effects of physical activity (Rebar et al., 2015; Stubbs et al., 2017; Chen and Nakagawa, 2023). Group psychological counseling, by contrast, may provide opportunities for guided reflection, peer interaction, role-playing, and communication practice, processes that have been discussed in previous literature on psychological interventions for college students (Lattie et al., 2019). From the perspective of Bandura’s social cognitive theory (Bandura, 1977), mastery experiences, vicarious learning, and verbal encouragement represent plausible pathways through which these intervention contexts might influence perceived self-efficacy. However, self-efficacy was not directly assessed in the present study, and this interpretation therefore remains theoretical.

The greater improvement observed in the DP-GC group may be consistent with a potentially additive benefit of integrating physical and psychological intervention components. Specifically, the DP-GC group showed significantly greater IAS improvement than both standalone groups. For SAD, the combined intervention significantly exceeded GC but did not differ significantly from DP after adjustment, whereas for LSAS, the DP-GC group showed significantly greater reductions than both DP and GC. Thus, the advantage of the combined intervention was outcome-dependent rather than uniformly demonstrated in every pairwise comparison. The SCL-90 findings further indicated broad reductions in psychological symptom scores; however, because the SCL-90 was used as a measure of general psychological distress rather than a social-anxiety-specific diagnostic instrument, these findings should be regarded as supportive rather than direct evidence of social anxiety-specific mechanisms.

Importantly, physiological arousal, emotional regulation, cognitive restructuring, executive function, self-confidence, social competence, and self-efficacy were not directly assessed as outcomes or mediating variables. Accordingly, the potential physiological, behavioral, and psychosocial pathways discussed above should be regarded as literature-informed hypotheses rather than mechanisms empirically demonstrated by the present study. In addition, structured intervention exposure was not dose-matched across the active treatment groups. Therefore, the stronger overall improvement observed in the combined condition cannot be attributed specifically to synergistic interaction between dancesport exercise and group psychological counseling. Dose-matched trials incorporating direct measures of candidate mediators are needed to distinguish additive treatment effects from specific mechanistic synergy.

### 4.1 Limitations

Despite the academically meaningful findings of the present study, several limitations should be acknowledged. First, all participants were recruited from a dancesport club at a single university, which may limit the generalizability of the findings to the broader population of Chinese college students. Although participants, including those in the control group, were instructed to avoid non-study dancesport activities, compliance and habitual physical activity were not objectively monitored; therefore, differences in actual physical activity exposure across groups cannot be excluded. The duration and frequency of participants’ prior dancesport experience were not systematically quantified, which may have influenced their responses to the intervention. In particular, the external validity of the results remains to be established among students with limited prior interest in or experience of dance-based physical activity. Second, the final sample size was relatively small, which may have reduced statistical power and affected the stability and reproducibility of the findings. Future studies should therefore recruit larger samples, preferably across multiple institutions, to improve the statistical reliability and representativeness of the results. In addition, the present study included only pre- and post-intervention assessments and did not incorporate medium- or long-term follow-up measurements. Consequently, the durability and long-term stability of the intervention effects could not be fully evaluated. Future research should address these limitations by increasing the sample size, broadening recruitment sources, incorporating additional follow-up assessment points, and including direct measures of potential physiological, cognitive, affective, and behavioral mechanisms. Such methodological refinements would strengthen the scientific rigor of the research and provide more robust evidence regarding the long-term effectiveness and broader applicability of the intervention strategies.

### 4.2 Future research directions

Future studies should recruit larger and more diverse samples from multiple universities across different regions of China to improve the representativeness and generalizability of the findings. Greater inclusion of students with little or no prior dancesport experience would also help determine whether the intervention effects can be extended to broader student populations. Methodological rigor should be further strengthened through preregistration, clearly defined primary outcomes, allocation concealment, and dose- or time-matched intervention designs. In addition, longitudinal follow-up assessments at 3, 6, and 12 months are needed to evaluate the durability and stability of intervention effects. Future research may also integrate self-report scales with behavioral observations, physiological indicators, and qualitative interviews to provide a more comprehensive understanding of the mechanisms through which dancesport exercise and group psychological counseling may influence social anxiety.

### 4.3 Practical implications

The findings of this study provide practical implications for university mental health promotion and social anxiety intervention. Dancesport may serve as an accessible and participatory physical activity-based approach that encourages student engagement and provides opportunities for repeated social interaction. Group psychological counseling, in contrast, may offer structured psychological support through cognitive, emotional, and interpersonal processes. Integrating these two approaches may therefore provide a broader and more flexible intervention framework for college students with social anxiety. Such programs could potentially complement existing campus mental health services and offer additional options for students who are less willing to participate in conventional counseling. Nevertheless, the apparent advantage of the combined intervention should be interpreted cautiously, and larger dose-matched studies with longer follow-up periods are required before its superiority can be established.

## 5 Conclusion

This randomized controlled trial provides preliminary evidence that dancesport exercise prescription and group psychological counseling were associated with reductions in social anxiety-related symptoms among Chinese university students. Both standalone interventions produced greater pre-to-post improvements than the control condition across several outcomes, although their relative effects varied across measures. The combined DP-GC intervention generally showed the largest reductions, particularly for interaction anxiety and overall social anxiety severity, suggesting a potential added benefit of integrating structured physical activity with group-based psychological support.

These findings support further investigation of integrated exercise- and counseling-based approaches as a potential intervention strategy for social anxiety in university settings. However, the observed advantage of the combined intervention should not be interpreted as definitive evidence of mechanistic synergy, particularly because structured intervention exposure was not dose-matched across the active conditions and the proposed physiological and psychological pathways were not directly assessed. Larger, preregistered, and adequately powered trials with dose-matched intervention conditions, longer follow-up, and direct assessment of potential mediating mechanisms are required to confirm the present findings and clarify the processes underlying intervention response.

## Data Availability

The raw data supporting the conclusions of this article will be made available by the authors, without undue reservation.
