# Supplementary material for: Social anxiety intervention for college students: the effect of Dancesport exercise prescriptions combined with group psychological counseling
Source: Front Psychol. 2026 Oct 7;17:1854500. doi: 10.3389/fpsyg.2026.1854500 (PMC13688642; doi:10.3389/fpsyg.2026.1854500)
Supplement: Supplementary file 1 [file Supplementary_file_1.docx]

**Social Anxiety Intervention for College Students: The Effect of Dancesport Exercise Prescriptions Combined with Group Psychological Counselling**

Mengmeng Yuan^1†^, Chen Jie^2,3†^, Dan Gong^4^, Shunli Wu^5^, Weikai Chen^6*^, Yanning Li^7*^

^1^School of Marxism, Huainan Normal University, Huainan, Anhui, China.

^2^School of Politics and Public Administration, Guangxi Normal University, Guilin, China.

^3^Department of Student Affairs, Jiaxing Vocational Technical College, Jiaxing, China.

^4^Chongqing College of International Business and Economics, College of Physical Education and Health, Chongqing, China.

^5^College of Marine Life Sciences, Ocean University of China, Qingdao, China.

^6^Department of Orthopedics, The Second Affiliated Hospital and Yuying Children’s Hospital of Wenzhou Medical University, Wenzhou, China.

^7^School of Physical Education, Yanshan University, Hebei, China.

**Supplementary Appendix A.**

### Dancesport Exercise Prescription

#### Supplementary Table A1. Weekly progression of the dancesport exercise prescription

| **Weeks** | **Warm-up activities** | **Main dance training content** | **Exercise intensity** | **Duration per session** |
| --- | --- | --- | --- | --- |
| 1–3 | Jogging and full-body stretching | Basic steps and posture training for Latin and Modern dance | Low to moderate intensity | 20 min warm-up + 30 min dance training + 15 min cool-down |
| 4–6 | Jogging/full-body stretching or skipping rope/dynamic stretching | Simple dance combinations and basic Waltz steps | Moderate intensity | 20 min warm-up + 35 min dance training + 15 min cool-down |
| 7–8 | Skipping rope and dynamic stretching | Basic Rumba movements, body lines, and rhythm training | Moderately high intensity | 20 min warm-up + 40 min dance training + 15 min cool-down |
| 9–10 | Skipping rope and dynamic stretching | Complex combinations and transitions in Rumba and Waltz | High intensity | 20 min warm-up + 45 min dance training + 15 min cool-down |
| 11–12 | Skipping rope and dynamic stretching | Complete dance performance practice and simulated competition scenarios | High intensity | 20 min warm-up + 50 min dance training + 15 min cool-down |

#### Note. The table summarizes the weekly progression of the dancesport exercise prescription, including training content, exercise intensity, and session duration.

#### Rationale for Progression The progression of the dancesport intervention was designed according to the principle of progressive overload and exercise-prescription safety. Training duration, movement complexity, and performance demands were gradually increased to allow participants to adapt physically and psychologically over the 12-week intervention. The protocol was developed by the research team and reviewed by experienced dancesport instructors to ensure feasibility and safety for college students with elevated social anxiety.

#### Exercise Intensity Monitoring and HRmax Determination Exercise intensity was monitored during each dancesport session using wearable heart-rate monitors. Participants’ heart rates were checked regularly to ensure that they remained within the prescribed target intensity range. When heart rates exceeded or fell below the target range, the instructor adjusted movement difficulty, tempo, or rest intervals accordingly. Maximal heart rate was estimated using the age-predicted formula: HRmax = 220 - age. The prescribed exercise intensity was then calculated as a percentage of the estimated HRmax.

#### I. Warm-Up Activities

The primary purpose of warm-up activities is to preheat the body, improve muscle elasticity and joint flexibility, and prepare for subsequent dance exercises.

1. Weeks 1-4

**Content**: 10 minutes of jogging, full-body stretching (including legs, waist, back, and arms)

**Exercise Intensity**: Low intensity, heart rate controlled at 50%-60% of maximum heart rate

**Duration**: 20 minutes

2. Weeks 5-12

**Content**: 5 minutes of skipping rope, dynamic stretching (including body twists, leg swings)

**Exercise Intensity**: Moderate intensity, heart rate controlled at 60%-70% of maximum heart rate

**Duration**: 20 minutes

#### II. Dance Exercise

Dance exercises are designed based on individual levels and goals, gradually enhancing dance skills and physical fitness.

1. Weeks 1-3

**Content**: Basic steps and posture training for Latin and Modern dance

**Exercise Intensity**: Low to moderate intensity, focusing on accuracy and coordination of movements

**Duration**: 30 minutes

2. Weeks 4-6

**Content**: Introduction of simple dance combinations, basic steps of Waltz

**Exercise Intensity**: Moderate intensity, gradually increasing continuity and speed of movements

**Duration**: 35 minutes

3. Weeks 7-8

**Content**: Learning and practicing basic movements of Rumba, emphasizing body lines and sense of rhythm

**Exercise Intensity**: Moderately high intensity, emphasizing rhythm and expressiveness of dance

**Duration**: 40 minutes

4. Weeks 9-10

**Content**: Training on complex combinations and transitions in Rumba and Waltz

**Exercise Intensity**: High intensity, focusing on the complexity and variability of movements

**Duration**: 45 minutes

5. Weeks 11-12

**Content**: Practice of complete dance performances, simulating competition scenarios

**Exercise Intensity**: High intensity, simulating dance performance under competition pressure

**Duration**: 50 minutes

#### III. Cool-Down Activities

Cool-down activities aim to relieve muscle tension and promote body recovery.

1. **Weeks 1-12**

**Content**: Deep breathing exercises, static stretching (focusing on relaxing thigh, calf, gluteal, and waist muscles)

**Exercise Intensity**: Low intensity, heart rate gradually returns to resting levels

**Duration**: 15 minutes

#### Notes:

Ensure correct breathing techniques during exercise to avoid holding your breath.

Adjust exercise intensity and dance difficulty as appropriate based on individual conditions to ensure safety and effectiveness of the exercise.

It is recommended to perform proper warm-up exercises before dance training to reduce the risk of exercise injuries.

Timely cool-down activities after dance training help alleviate muscle fatigue and prevent exercise injuries.

### Supplementary Appendix B.

### Group Psychological Counselling Protocol

#### I. Overview of Group Stages and Goals

This group psychological counselling intervention programme is divided into four stages: initial stage (building trust and team foundation), transition stage (deepening self-awareness and building confidence), working stage (enhancing self-acceptance, communication skills, and adaptability), and concluding stage (summary review and future planning). Through carefully designed 12-week activities, the programme aims to help college students comprehensively improve their psychological quality, enhance self-awareness, confidence, communication and cooperation abilities, and adaptability to changes.

**II. Session Structure and Time Allocation**
Each group psychological counselling session lasted approximately 100 minutes. Each session included opening and emotional check-in, 10 minutes; psychoeducation or theme introduction, 15 minutes; structured group activity or role-playing, 35 minutes; group discussion and guided reflection, 25 minutes; and feedback, summary, and between-session practice assignment, 15 minutes. Minor adjustments were allowed according to the weekly theme and group dynamics.

#### III. Weekly Group Psychological Counselling Sessions

#### Week 1: Getting Acquainted and Team Building

**Goal**: Members get to know each other and build team cohesion.

**Content**:

Self-introductions and ice-breaking games such as "Name Chain" or "Quick Questions."

Explanation of the purpose, significance, and rules of group psychological counselling, and clarification of common goals.

Team-building activities like "Building Blocks" or "Team Puzzle" to enhance team cooperation awareness.

#### Week 2: Emotional Expression and Listening

**Goal**: Learn to effectively express emotions and improve listening skills.

**Content**:

Teaching and practicing emotional expression techniques, such as using "I-statements" to express feelings.

Training in listening skills, such as active responding, not interrupting, and providing feedback on feelings.

Emotional sharing time, where members take turns sharing personal stories and listening to each other.

#### Week 3: Self-Awareness and Acceptance

**Goal**: Deepen self-understanding and accept both strengths and weaknesses.

**Content**:

Self-exploration activities, such as filling out self-awareness questionnaires and analyzing personal characteristics.

Clarification of values, identifying core personal values, and life goals.

Self-acceptance exercises, like writing a self-acceptance letter to encourage personal growth.

#### Week 4: Interpersonal Relationships and Communication Skills

**Goal**: Improve interpersonal skills and learn effective communication techniques.

**Content**:

Explanation of the importance of interpersonal relationships and analysis of common issues in relationships.

Training in communication skills, such as nonviolent communication and emotional listening.

Role-playing to simulate communication in different scenarios, enhancing practical skills.

#### Week 5: Emotion Recognition and Management

**Goal**: Recognize and manage emotions, enhancing emotional intelligence.

**Content**:

Emotion recognition games or tests to help members understand their emotional types.

Introduction of emotion management strategies, such as deep breathing, emotion diaries, and meditation.

Emotion sharing and discussion, where members share emotion management experiences and learn from each other.

#### Week 6: Stress Coping and Psychological Adjustment

**Goal**: Learn to cope with stress and maintain psychological balance.

**Content**:

Identification and discussion of stress sources, analyzing personal stress origins.

Explanation of stress coping strategies, such as time management, relaxation training, and seeking support.

Relaxation exercises like progressive muscle relaxation or yoga to help members relax their bodies and minds.

#### Week 7: Confidence Building and Self-Motivation

**Goal**: Enhance self-confidence and learn self-motivation techniques.

**Content**:

Explanation of the importance of confidence and analysis of causes of lack of confidence.

Introduction of confidence-building methods, such as self-affirmation and setting small goals.

Self-motivation exercises, like creating "Success Cards" or "Self-Motivation Quotes" to stimulate inner motivation.

#### Week 8: Conflict Resolution and Promoting Cooperation

**Goal**: Learn to resolve conflicts and promote team cooperation.

**Content**:

Analysis of conflict types and impacts, understanding the universality of conflicts.

Explanation of conflict resolution strategies, such as win-win strategies, compromise, and finding common ground.

Team cooperation games or projects simulating conflict scenarios to practice conflict resolution techniques.

#### Week 9: Goal Setting and Planning

**Goal**: Set personal goals and develop implementation plans.

**Content**:

Explanation of the importance of goal setting to stimulate member enthusiasm.

Introduction of the SMART goal-setting method to guide members in setting specific, measurable goals.

Development and sharing of action plans, with members providing feedback and suggestions to each other.

#### Week 10: Coping with Setbacks and Building Resilience

**Goal**: Learn to cope with setbacks and develop psychological resilience.

**Content**:

Explanation of the universality and inevitability of setbacks and their impact on personal growth.

Introduction of setback coping strategies, such as adjusting mindset, seeking support, and summarizing lessons learned.

Sharing and discussing experiences and insights on coping with setbacks to enhance psychological resilience.

#### Week 11: Emotional Support and Peer Assistance

**Goal**: Enhance emotional support and foster a spirit of peer assistance.

**Content**:

Explanation of the importance of emotional support and its impact on mental health.

Training in peer assistance techniques, such as active listening, providing advice, and offering encouragement.

Emotional support practice session, where members provide emotional support to each other, building deep friendships.

#### Week 12: Farewell and Future Outlook

**Goal**: Review the journey, look ahead to the future, and establish long-term connections.

**Content**:

Review the entire process and achievements of the group psychological counselling.

Sharing personal growth changes and insights.

Discussing future plans and goals, and making future plans.

**Additional Methodological Information**

**Intervention Adherence Criteria and Monitoring**
Intervention adherence was defined as attendance at ≥80% of the scheduled intervention sessions. Attendance was recorded after each session by the facilitator responsible for the intervention. Adherence was calculated as the number of attended sessions divided by the total number of scheduled sessions. Missed sessions, withdrawals, and protocol deviations were recorded throughout the 12-week intervention.

**Assessment Procedures**
All psychological instruments were self-administered by participants in a standardized group setting under the supervision of trained personnel who had received psychological training and obtained National Level-2 Psychological Counselor certification. Before data collection, these personnel received standardized instructions on questionnaire administration, confidentiality requirements, item clarification, and data checking. They did not provide psychological interpretation or intervention during assessment.

**Rationale for Instrument Selection**
The four instruments were selected to assess related but distinct dimensions of social anxiety and associated psychological distress. The IAS was used to assess subjective interaction anxiety and cognitive-affective unease in interpersonal situations. The SAD was used to assess social avoidance and distress, with emphasis on behavioural avoidance and discomfort in social contexts. The LSAS was used to assess the broader severity of anxiety and avoidance across social and performance situations. The SCL-90 was included as an auxiliary measure of broader psychological distress and related symptom dimensions. Together, these instruments allowed the study to evaluate both core social anxiety symptoms and associated psychological changes.

**Supplementary Appendix C**

Symptom Checklist 90(SCL-90)

Name: Gender: Grade:

Instructions:The following is a list of questions that some people may have. Please read each one carefully, and then indicate the degree of score after each question based on how the following situation affected your actual feeling within the last week. Select 1 for "None", 2 for "Very light", 3 for "Medium", 4 for "heavy", and 5 for "severe".

| **Serial number** | **Questionnaire entry** | **option** |
| --- | --- | --- |
| 1 | Headaches. |  |
| 2 | You're nervous and insecure. |  |
| 3 | You have unnecessary thoughts or words swirling in your head. |  |
| 4 | Dizziness or fainting. |  |
| 5 | Decreased interest in the opposite sex. |  |
| 6 | Blaming yourself for things. |  |
| 7 | Feeling that someone else can control your thoughts. |  |
| 8 | Blame others for causing problems. |  |
| 9 | It is easy to forget. |  |
| 10 | Worry about their neat clothes and good manners. |  |
| 11 | You get upset and excited easily. |  |
| 12 | Chest pain. |  |
| 13 | Fear of empty Spaces or streets. |  |
| 14 | You feel your energy levels drop and your activities slow down. |  |
| 15 | Want to end your life. |  |
| 16 | Hearing sounds that others can't hear. |  |
| 17 | Shivering. |  |
| 18 | Feeling that most people can't be trusted. |  |
| 19 | Poor appetite. |  |
| 20 | You cry easily. |  |
| 21 | Feeling shy and uncomfortable around the opposite sex. |  |
| 22 | Feeling cheated, snared or someone trying to catch you. |  |
| 23 | Sudden fear for no apparent reason. |  |
| 24 | You lose your temper uncontrollable. |  |
| 25 | Fear of going out alone. |  |
| 26 | Constantly blaming yourself. |  |
| 27 | Lower back pain. |  |
| 28 | Feeling difficult to complete tasks. |  |
| 29 | Feeling lonely. |  |
| 30 | Feeling miserable. |  |
| 31 | Worrying too much. |  |
| 32 | Disinterest in things. |  |
| 33 | Feeling scared. |  |
| 34 | Your feelings are easily hurt. |  |
| 35 | People can know your private thoughts. |  |
| 36 | Feeling that others don't understand or sympathize with you. |  |
| 37 | Feeling that people aren't nice to you or like you. |  |
| 38 | Things must be done slowly to be done correctly. |  |
| 39 | My heart beats very hard. |  |
| 40 | Nausea or upset stomach. |  |
| 41 | Feeling inferior to others. |  |
| 42 | Sore muscles. |  |
| 43 | Feeling that someone is watching you and talking about you. |  |
| 44 | Difficulty falling asleep. |  |
| 45 | Things must be checked and rechecked. |  |
| 46 | It is difficult to make a decision. |  |
| 47 | Fear of trams, buses, subways or trains. |  |
| 48 | Have difficulty breathing. |  |
| 49 | Bouts of chills or fever. |  |
| 50 | Avoiding things, situations, or activities because you are afraid. |  |
| 51 | The mind is empty. |  |
| 52 | Numbness or tingling in the body. |  |
| 53 | feeling a blockage in my throat |  |
| 54 | Feeling hopeless about the future. |  |
| 55 | Inability to concentrate. |  |
| 56 | Feeling weak in a part of your body. |  |
| 57 | Feeling nervous or easily nervous. |  |
| 58 | Feeling heaviness in your arms or legs. |  |
| 59 | Thinking about death. |  |
| 60 | You eat too much. |  |
| 61 | Feeling uncomfortable when people look at you or talk about you. |  |
| 62 | Have some ideas that are not your own. |  |
| 63 | The urge to hit or hurt others. |  |
| 64 | Waking up too early. |  |
| 65 | You must repeatedly wash your hands, count or touch something. |  |
| 66 | Sleep unsteadily and deeply. |  |
| 67 | The urge to break or destroy things. |  |
| 68 | Having thoughts or ideas that others don't have. |  |
| 69 | Feeling nervous about other people |  |
| 70 | Feeling uncomfortable in crowded places  such as shops or cinemas. |  |
| 71 | Feeling that everything is difficult. |  |
| 72 | Bouts of fear or panic. |  |
| 73 | I feel uncomfortable eating in public. |  |
| 74 | Often argue with others. |  |
| 75 | You're nervous when you're alone |  |
| 76 | Your achievements have not been properly evaluated. |  |
| 77 | You feel lonely even when you're with others. |  |
| 78 | Feeling restless and unsettled. |  |
| 79 | Feeling worthless. |  |
| 80 | Something that feels familiar becomes strange or unreal. |  |
| 81 | Yelling or throwing things. |  |
| 82 | Fear of passing out in public. |  |
| 83 | Feeling that someone is trying to take advantage of you. |  |
| 84 | Suffering from sexual thoughts. |  |
| 85 | You believe that you should be punished for your own mistakes |  |
| 86 | Feeling the need to get things done quickly. |  |
| 87 | Feeling that there is something seriously wrong with your body. |  |
| 88 | Never felt close to anyone else. |  |
| 89 | Feeling guilty. |  |
| 90 | Feeling that something is wrong with your head. |  |

**Supplementary Appendix D**

Interaction Anxiousness Scale（IAS）

Name：	Gender：	Age:

Instructions:Please read each of the following entries carefully and decide the extent to which their statements apply to you or are true. Mark the score in the space before the corresponding entry according to the following criteria (1-5).(1=This article does not agree with me at all, 2=this article agrees with me at all,3=This article agrees with me moderately, 4=This article agrees with me very well,5=This article agrees with me very much)

| **Serial number** | **Questionnaire entry** | 1 | 2 | 3 | 4 | 5 |
| --- | --- | --- | --- | --- | --- | --- |
| 1 | I often feel nervous even at informal parties |  |  |  |  |  |
| 2 | I usually feel out of place when I'm with a group of people |  |  |  |  |  |
| 3 | I usually feel relaxed when talking to a person of  the opposite sex. (R) |  |  |  |  |  |
| 4 | I feel nervous when I have to talk to my teacher or boss. |  |  |  |  |  |
| 5 | Parties often make me feel anxious and uncomfortable. |  |  |  |  |  |
| 6 | I may be less shy in social interactions than most people (R) |  |  |  |  |  |
| 7 | I often feel nervous when talking to people of the same sex whom I don't know very well. |  |  |  |  |  |
| 8 | I get nervous during job interviews. |  |  |  |  |  |
| 9 | I wish I had more confidence in social situations |  |  |  |  |  |
| 10 | I seldom feel anxious in social situations. (R) |  |  |  |  |  |
| 11 | Generally speaking, I am a shy person. |  |  |  |  |  |
| 12 | I often feel nervous when talking to an attractive person of  the opposite sex. |  |  |  |  |  |
| 13 | I usually feel nervous when calling people  I don't know very well. |  |  |  |  |  |
| 14 | I feel nervous when talking to authority figures. |  |  |  |  |  |
| 15 | I usually feel relaxed even when I am in a group of people quite different from me. (R) |  |  |  |  |  |

**Supplementary Appendix E**

Social Avoidance and Distress Scale (SAD)

Name：	Gender：	Age:

Instructions:Here are some questions you need to answer, please choose the most correct answer according to your actual situation and reaction. (1, yes 2, no)

| **Serial number** | **Questionnaire entry** | **Yes** | **No** |
| --- | --- | --- | --- |
| 1 | I feel relaxed even in unfamiliar social situations |  |  |
| 2 | I try to avoid situations that force me  to take part in social activities |  |  |
| 3 | I relax easily when I'm with strangers |  |  |
| 4 | I don't particularly want to avoid people |  |  |
| 5 | I usually find social situations frustrating |  |  |
| 6 | I usually feel calm and comfortable in social situations |  |  |
| 7 | I usually feel relaxed when talking to the opposite sex |  |  |
| 8 | I try to avoid talking to people unless I know them very well |  |  |
| 9 | If there's an opportunity to meet someone new, I'll take it |  |  |
| 10 | I usually feel anxious and uneasy at informal gatherings  when the opposite sex is present |  |  |
| 11 | I usually feel anxious around people unless  I know them very well |  |  |
| 12 | I usually feel relaxed when I'm with a group |  |  |
| 13 | I often want to get away from crowds |  |  |
| 14 | I feel uncomfortable around people I don't know |  |  |
| 15 | I'm usually relaxed when I meet someone for the first time |  |  |
| 16 | Being introduced to others makes me nervous and anxious |  |  |
| 17 | I might go in even though the room is full of strangers |  |  |
| 18 | I avoid walking up and joining large groups of people |  |  |
| 19 | I am happy to talk to my boss when he wants to talk to me |  |  |
| 20 | I usually feel butterflies in my stomach when  I'm with a group of people |  |  |
| 21 | I like to avoid people |  |  |
| 22 | I have no problem talking to people in the  evening or at social gatherings |  |  |
| 23 | I rarely feel at home in large groups of people |  |  |
| 24 | I often come up with excuses to avoid social activities |  |  |
| 25 | I sometimes act as an introduction to people |  |  |
| 26 | I try to avoid formal social situations |  |  |
| 27 | I usually take part in every social interaction I can. |  |  |
| 28 | I find it easy to relax when I'm with others |  |  |

**Supplementary Appendix F**

Liebowitz Social Anxiety Scale（LSAS）

Name：	Gender：	Age:

Instructions:Please read the following questions carefully and judge the extent to which they correspond to your feelings and actions. From options 1 to 4, choose the one you actually feel, and then from options 5 to 8, choose the one you actually act in. (Fear/anxiety: 1, no 2, mild 3, severe 4, serious.avoidance: 5, never (0%) 6, occasionally (1-33%) 7, Frequently (34-67%) 8, often (68-100%))

| **Serial**  **number** | **Questionnaire entry** | **Fear/anxiety** | | | | **avoidance** | | | |
| --- | --- | --- | --- | --- | --- | --- | --- | --- | --- |
|  |  | **none** | **mild** | **moderate** | **severe** | **never** | **occasionally** | **often** | **usually** |
| 1 | Making phone calls in public |  |  |  |  |  |  |  |  |
| 2 | Participate in group activities |  |  |  |  |  |  |  |  |
| 3 | Eating in public |  |  |  |  |  |  |  |  |
| 4 | Drinking in public |  |  |  |  |  |  |  |  |
| 5 | Talk to important people |  |  |  |  |  |  |  |  |
| 6 | Perform, present, or speak in front of an audience |  |  |  |  |  |  |  |  |
| 7 | Attend a party |  |  |  |  |  |  |  |  |
| 8 | Work with someone watching |  |  |  |  |  |  |  |  |
| 9 | Being watched while you write |  |  |  |  |  |  |  |  |
| 10 | Make phone calls with people you don't know very well |  |  |  |  |  |  |  |  |
| 11 | Talk to people you don't know very well |  |  |  |  |  |  |  |  |
| 12 | Meeting strangers |  |  |  |  |  |  |  |  |
| 13 | Urinating in a public bathroom |  |  |  |  |  |  |  |  |
| 14 | Enter a seated room |  |  |  |  |  |  |  |  |
| 15 | Be the center of attention |  |  |  |  |  |  |  |  |
| 16 | Address the conference |  |  |  |  |  |  |  |  |
| 17 | Take the test |  |  |  |  |  |  |  |  |
| 18 | Express different opinions and opinions to people you don't know well |  |  |  |  |  |  |  |  |
| 19 | Make eye contact with people you don't know well |  |  |  |  |  |  |  |  |
| 20 | Debrief in group |  |  |  |  |  |  |  |  |
| 21 | Try to connect with someone |  |  |  |  |  |  |  |  |
| 22 | Go to the store to return the item |  |  |  |  |  |  |  |  |
| 23 | Organize parties |  |  |  |  |  |  |  |  |
| 24 | Refuse a hard sell from a salesman |  |  |  |  |  |  |  |  |
